# Relationships between stable isotope natural abundances (δ^13^C and δ^15^N) and water use efficiency in rice under alternate wetting and drying irrigation in soils with high clay contents

**DOI:** 10.3389/fpls.2022.1077152

**Published:** 2022-12-02

**Authors:** Zhenchang Wang, Jinjing Liu, Yaosheng Wang, Evgenios Agathokleous, Yousef Alhaj Hamoud, Rangjian Qiu, Cheng Hong, Minghao Tian, Hiba Shaghaleh, Xiangping Guo

**Affiliations:** ^1^ College of Agricultural Science and Engineering, Hohai University, Nanjing, China; ^2^ Jiangsu Province Engineering Research Center for Agricultural Soil-Water Efficient Utilization, Carbon Sequestration and Emission Reduction, Nanjing, China; ^3^ Institute of Environment and Sustainable Development in Agriculture, Chinese Academy of Agricultural Sciences, Beijing, China; ^4^ School of Applied Meteorology, Nanjing University of Information Science and Technology, Nanjing, China; ^5^ Department of Soil and Land Reclamation, Aleppo University, Aleppo, Syria; ^6^ State Key Laboratory of Water Resources and Hydropower Engineering Science, Wuhan University, Wuhan, China; ^7^ College of Environment, Hohai University, Nanjing, China

**Keywords:** alternate wetting and drying irrigation, soil clay content, δ^13^C, δ^15^N, water use efficiency, *Oryza sativa L*

## Abstract

Natural abundance of the stable isotope (δ^13^C and δ^15^N) in plants is widely used to indicate water use efficiency (WUE). However, soil water and texture properties may affect this relationship, which remains largely elusive. Therefore, the purpose of this study was to evaluate δ^13^C as affected by different combinations of alternate wetting and drying irrigation (AWD) with varied soil clay contents in different organs and whole plant and assess the feasibility of using δ^13^C and δ^15^N as a physiological indicator of whole-plant water use efficiency (WUE_whole-plant_). Three AWD regimes, I_100_ (30 mm flooded when soil reached 100% saturation), I_90_ (30 mm flooded when reached 90% saturation) and I_70_ (30 mm flooded when reached 70% saturation) and three soil clay contents, 40% (S_40_), 50% (S_50_), and 60% (S_60_), were included. Observed variations in WUE_whole-plant_ did not conform to theoretical expectations of the organs δ^13^C (δ^13^C_organs_) of plant biomass based on pooled data from all treatments. However, a positive relationship between δ^13^C_leaf_ and WUE_ET_ (dry biomass/evapotranspiration) was observed under I_90_ regime, whereas there were no significant relationships between δ^13^C_organs_ and WUE_ET_ under I_100_ or I_70_ regimes. Under I_100_, weak relationships between δ^13^C_organs_ and WUE_ET_ could be explained by (i) variation in C allocation patterns under different clay content, and (ii) relatively higher rate of panicle water loss, which was independent of stomatal regulation and photosynthesis. Under I_70_, weak relationships between δ^13^C_organs_ and WUE_ET_ could be ascribed to (i) bigger cracks induced by water-limited irrigation regime and high clay content soil, and (ii) damage caused by severe drought. In addition, a negative relationship was observed between WUE_whole-plant_ and shoot δ^15^N (δ^15^N_shoot_) across the three irrigation treatments, indicating that WUE_whole-plant_ is tightly associated with N metabolism and N isotope discrimination in rice. Therefore, δ^13^C should be used cautiously as an indicator of rice WUE_whole-plant_ at different AWD regimes with high clay content, whereas δ^15^N could be considered an effective indicator of WUE_whole-plant_.

## Introduction

As one of the world’s most widely cultivated crops, rice provides calories for half the world’s population (Runkle et al., 2021). Nearly two-thirds of the total rice production depends on flooded irrigation (Umesh et al., 2018). However, water for agriculture is facing increasing challenges due to land degradation, water scarcity, chemical contamination, and extreme weather caused by climate changes (Kümmerer et al., 2018). Since crop yield losses due to water deficit is greater than those attributed to other environmental stressors worldwide, efficient use of water resources is of paramount significance (Gao et al., 2018). At leaf level, water use efficiency (WUE) is often defined as the ratio between carbon fixation (A_n_) and stomatal conductance to water vapor (g_s_) (so-called intrinsic WUE, viz WUE_i_). The WUE at whole-plant level (WUE_whole-plant_) is usually represented by three functions: (i) WUE_T_=Total dry biomass/transpiration (T) (Topbjerg et al., 2014), (ii) WUE_ET_=Total dry biomass/evapotranspiration (ET), and (iii) WUE_I_=Total dry biomass/irrigation amount (I) (Gao et al., 2018). The WUE based on transpiration contains information about plant photosynthesis with water physiological processes and seems to be most relevant for crop physiological traits (Impa et al., 2005). The WUE_ET_ and WUE_I_ are mainly determined from dry matter production and soil water loss (relating to transpiration and soil surface evaporation for WUE_ET_, and relating to transpiration, soil surface evaporation as well as seepage and percolation of soil for WUE_I_, respectively). Therefore, the WUE_ET_ and WUE_I_ can be used as an integrated indicator of environmental conditions affecting plant water relations and dry matter production (Zhao et al., 2004).

Instead of traditional flooded irrigation of rice, an efficient water–saving practice, called alternate wetting and drying (AWD) irrigation, has been widely used. This irrigation practice introduces unsaturated soil conditions into the irrigation scheduling during the growing season, which allows a reduction in the water layer depth until the soil is slightly dry before the next irrigation (Oliver et al., 2019). Numerous studies have demonstrated that AWD improves WUE_whole-plant_ by 35–63% (Reis et al., 2018; Oliver et al., 2019; Haque et al., 2021; Song et al., 2021). However, several studies reported that the low soil water potential during drying stage of AWD would adversely affect crop physiology and growth, eventually reducing the yield and exhibiting a low WUE_whole-plant_ (Walley et al., 1999; Carrijo et al., 2017). In addition, seepage and evapotranspiration vary with different statuses of water and soil in the field, for instance, seepage values during cultivated stage were observed to be as high as 25 mm day^−1^ due to soil cracks in rice fields (Datta et al., 2017). Therefore, it remains debatable whether WUE is improved under AWD, especially in different soil types.

Soil clay content is another factor that can directly or indirectly affect WUE. Clay content affects the ability of soil to retain carbon (C), water, and nutrient ions, thus affecting the biophysiochemical processes in plants (Silver et al., 2000). The interaction between soil texture and irrigation regime is complex. Soil with high clay content expend and swell periodically under AWD in paddy fields, easily causing cracks whose volumes are affected by the severity of the drought (Al-Jeznawi et al., 2020; Bordoloi et al., 2020). The existence of cracks can promote the evaporation rate through the increased soil–air interface, aggravating the damage of drought (Cheng et al., 2021). Moreover, cracks offer a preferential flow path (closely related to seepage and percolation) in subsequent irrigation, accelerating water and fertilizer infiltration and influencing plant physiological responses, hence indirectly reducing yield and WUE (Wang et al., 2018; Cheng et al., 2021). Meanwhile, the tearing effect of cracks on the root system under the high clay content will also affect the plant dry matter allocation and physiological processes, therefore affecting WUE (Ma et al., 2008; Ren et al., 2021). Due to the influence of cracks and soil texture, soil with different clay contents not only differs in evaporation and leakage, but also leads to varied plant physiological responses, such as photosynthesis, transpiration, and C transfer and allocation (Awale et al., 2013; Dou et al., 2016). Therefore, the responses of varying types of WUE, such as WUE_T_, WUE_ET_, and WUE_I_, to AWD regimes may differ in the presence of different clay contents.

The theory linking δ^13^C and WUE has been well established, and the physiological basis of such relationship is also well understood. Plants are known to vary in their discrimination against heavy isotope of carbon during the assimilation process of CO_2_ by photosynthetic carboxylase (30‰) and the diffusion process of atmospheric CO_2_ through stomata into leaves (4.4‰) (Domergue et al., 2022). Therefore, environmental factors affecting any of the A_n_ and g_s_ could have a direct effect on δ^13^C in plants (Livingston et al., 1999). For example, under drought condition, the isotope discrimination for ^13^C is relatively low, leading to an enriched ^13^C in plant matter (Domergue et al., 2022). As a result, there exists a positive linear relationship between δ^13^C and WUE_i_ in many crop species under drought conditions. Since WUE_whole-plant_ is highly related to WUE_i_, the δ^13^C in plant dry matter is supposed to be strongly correlated with the WUE_whole-plant_ (Blankenagel et al., 2022).

Although several studies found that δ^13^C in leaf is positively correlated with WUE_whole-plant_ under a certain degree of water deficit (Gouveia et al., 2019a; Mininni et al., 2022), the change of WUE_whole-plant_ is not always consistent with the change of plant δ^13^C under different irrigation regimes (Walley et al., 1999; Zhao et al., 2004). For instance, Zhao et al. (2004) found that δ^13^C in rice leaf at different growth periods was negatively correlated or even uncorrelated with WUE under varied irrigation regimes. Under a slight degree of water deficit for AWD, the cracks are probably small or do not exist (Alhaj Hamoud et al., 2018), while high clay content may suggest a changed stomatal conductance or photosynthetic capacity through the improved water and nitrogen condition in soil (Silver et al., 2000), eventually affecting δ^13^C value in plants. Nonetheless, under severe degree of water deficit for AWD, the soil with high clay content may strongly swell and shrink, leading to the formation of cracks (Al-Jeznawi et al., 2020). This could tear roots and influence the biomass allocation of plant organs (Silver et al., 2000), hence in turn it may affect δ^13^C in plant dry matter. However, the effect of different irrigation regimes as a function of varied soil clay contents on δ^13^C remains unknown. Additionally, the isotopic signatures of individual organs are more readily available for physiological and biochemical analysis compared to the whole-plant. The question thus arises as to whether δ^13^C_organs_ can be used to predict information on δ^13^C_whole-plant_. In particular, the internal partitioning and metabolism of primary assimilation may generate δ^13^C differences between plant organs (Robinson et al., 2000). Therefore, we hypothesized that different AWD regimes under varied soil clay contents could influence the C allocation and δ^13^C values in various organs, by influencing the soil condition such as water and fertilizer. The relationship between δ^13^C_organ_ and δ^13^C_whole-plant_, and between δ^13^C_organ_ and WUE_whole-plant_ as a function of varied soil clay contents might be modified accordingly.

In addition to δ^13^C, the N isotope composition (δ^15^N) of plant was also reported as a physiological indicator responding to drought stress conditions (Ulrich et al., 2019). The WUE_whole-plant_ is closely related to the WUE_i_ (A_n_/g_s_) of leaves as previously discussed (Blankenagel et al., 2022). Hence, any factor influencing A_n_ and/or *g_s_* would have a direct effect on WUE_i_, in turn affecting WUE_whole-plant_. It was reported that WUE_i_ was positively correlated with leaf nitrogen concentration ([N]_leaf_) under well-watered conditions (Topbjerg et al., 2014; Tang et al., 2017). Considering the variation of [N]_leaf_ and δ^15^N in plants were both closely linked to N metabolism in plants (Yousfi et al., 2013), it is suggested that there is a link between WUE_whole-plant_ and δ^15^N in plants. Consistent with this, Yousfi et al. (2012) showed that leaf δ^15^N was negatively correlated with transpiration efficiency in durum wheat exposed to salinity and water deficit. A similar relationship between δ^15^N_leaf_ and WUE has also been reported in potato (Topbjerg et al., 2014). However, Cao et al. (2014) revealed that WUE was positively correlated with δ^15^N in poplar (*Populus*) genotypes. The reasons for this discrepancy remain unknown. In addition, different AWD regimes under varied soil clay contents could also affect N metabolism as well as [N]_leaf_ and δ^15^N in different organs by influencing soil water and fertilizer conditions, therefore it might alter the relationship between the δ^15^N and WUE, which, however, also remains unclear.

In this study, an experiment was conducted to investigate the C allocation and ^13^C distribution in various organs of rice plants as influenced by different irrigation regimes and clay contents. We hypothesized that different irrigation regimes and clay contents could lead to changes in g_s_ and A_n_ of rice, thus affecting changes in WUE, δ^13^C, and δ^15^N. The combined application of different irrigation regimes and soil clay contents may cause C allocation changes, affecting the relationship between WUE and δ^13^C, as well as between δ^13^C_organ_ and δ^13^C_whole-plant_. Therefore, the main objectives of this study were to (1) investigate the effect of clay content on carbon allocation of rice organs under three irrigation regimes; (2) evaluate the effect of different irrigation regimes and clay contents on WUE and δ^13^C at the organ and the whole-plant levels, and (3) comprehensively analyze the relationships between (i) WUE and δ^13^C_organ_, (ii) WUE and δ^15^N_organ_, and (iii) δ^13^C_whole-plant_ and δ^13^C_organ_.

## Materials and methods

### Experimental site

The experiment was conducted at the Experimental Farm of the Soil and Water Engineering Department of Hohai University, Nanjing, China (longitude 118°83′E and latitude 31°95′N) during July and October, 2016. The area has a typical humid subtropical monsoon climate with an annual precipitation of 1062 mm. The mean temperature is 15.5°C. The used cylindrical pots were 51 cm in height and had a 16 cm inner diameter. Each pot was firstly filled with 1.2 kg gravel-sand soil at the bottom and then covered with 8 kg of dry soil. A drainage hole at the bottom of each pot and a movable basin under the pot were used to collect percolation water. Detailed information about the experimental pots was reported in Wang et al. (2022).

### Experimental design

The experiment had nine treatments, consisting of three water regimes and three soil clay contents. Each treatment was replicated four times. The pots were placed under a plastic shelter on a randomized complete block design. For the three water treatments, the pots maintained 25 mm of water over 7 d after transplanting to ensure plant establishment. After that, the upper limit in all the treatments was set as 30 mm flooding water, and the lower limits were 100%, 90%, and 70% of saturated soil water content, respectively (denoted as I_100_, I_90_, and I_70_, respectively). The specific irrigation process in this study is shown in Wang et al. (2022). The soil treatment was controlled using three different soil clay contents, i.e. 40%, 50%, and 60% (denoted as S_40_, S_50_ and S_60_, respectively). The original soil (i.e. S_40_) had a sand, silt, and clay fraction of 20.81%, 38.94%, and 40.25%, respectively. The S_50_ and S_60_ treatments were formed by mixing with respective amounts of pure clay. The selected physicochemical properties and corresponding measurements of soil are same as reported in Wang et al. (2022).

Two seedlings of rice (*Oryza sativa* L cv. Nanjing44) were transplanted in each pot on 20 July, 2016. Potassium phosphate (0.10 g P kg^−1^ soil) and potassium sulfate (0.13 g K kg^−1^ soil) were applied and incorporated before transplanting. In addition, all pots were fertilized with urea (0.15 g N kg^−1^ soil) in a four-split-application during vegetative and reproductive growth stages.

### Soil water content and soil crack volume

The original pot weight with dry soil was recorded. The gravimetrical soil moisture content was measured by weighing the pots based on weight loss every day:


(1) Soil moisture content = (wet soil - dry soil)/dry soil ×100%

The length, depth, and width of soil cracks were recorded by a steel rule with a 2mm diameter steel rod when the soil water content reached the lower limit of irrigation (100%, 90%, 70% saturated moisture respectively) before each irrigation event. The soil crack volume was calculated by assuming triangular shape of the cracks (Bandyopadhyay et al., 2003):


(2) V=∑0.5×dwl

where d, w and l are the depth, width, and length of the crack (cm), respectively.

The data of average crack volume was shown in **Supplementary Figure 1**.

### Plant sampling and measurements

#### SPAD, panicle length and dry biomass

The relative chlorophyll content (SPAD) was estimated with a portable chlorophyll meter (SPAD-502, Konica Minolta, Japan). All SPAD readings were taken at the middle portion of fully expanded flag leaf of rice at the full heading stage and were determined between 08:00 h and 11:00 h on a sunny day. The SPAD values of each pot were the average readings of five randomly selected flag leaves. After harvest of rice, the panicle length was measured by a ruler. In addition, grain, stem, leaf, and root samples were separately collected in paper bags. All samples were oven dried at 70°C for 72 h to a constant weight to measure the dry biomass.

#### Carbon concentration, carbon and nitrogen isotopic composition

After weighing, the rice samples were ground into a fine powder and sieved (2mm), and 0.1 g of fine powdered rice organs was used for measurement of isotopic composition. Carbon concentration([C], %), carbon isotopic composition (δ^13^C, ‰) and nitrogen isotopic composition (δ^15^N, ‰) in plant organs were determined using an Elemental Analyzer System (vario PYRO cube, Elementar Analysensysteme GmbH, Germany) interfaced with an Isotope Mass Spectrometer (Isoprime 100, Elementar Analysensysteme GmbH, Germany). The carbon content in organ (C_organ_) was calculated from the [C]_organ_ and the dry biomass of the respective organ. The whole plant C content (C_whole-plant_) was calculated from the C_grain_, C_stem_, C_leaf_, and C_root_. The C allocation (%) of organ was defined as the ratio of C_organ_ to C_whole-plant_. The δ^13^C and δ^15^N value of rice organs can be calculated as:


(3) $$\delta ‰=\left[\;\left(\frac{R_{sample}}{R_{standard}}\right)-1\right]\times 1000$$

where R is the ratio of ^13^C/^12^C or ^15^N/^14^N.

The δ^13^C _whole-plant_ was calculated as follows:


(4) $${\text{δ}}^{13}{\text{C}}_{whole-plant}\text{‰=}\frac{{\text{δ}}^{13}{\text{C}}_{\text{grain}}{\times \text{C}}_{grain}{\text{+δ}}^{13}{\text{C}}_{\text{stem}}{\times \text{C}}_{stem}+{\text{δ}}^{13}{\text{C}}_{\text{leaf}}{\times \text{C}}_{leaf}+{\text{δ}}^{13}{\text{C}}_{\text{root}}{\times \text{C}}_{root}}{{\text{C}}_{whole-plant}}$$

#### Evapotranspiration and WUE

The amounts of irrigation and percolation water were measured after each irrigation event and were shown in **Supplementary Figure 2**. The total evapotranspiration (ET) over the growing season for each pipe was determined as the summation of difference between total irrigation water volume and percolation water volume. The accumulative transpiration was calculated by subtracting the water evaporation from the evapotranspiration, as shown in **Supplementary Figure 2**. The detailed measurement of the surface evaporation loss was displayed in **Supplementary information**. Water use efficiencies (WUE_s_) were computed as


(5) $${\text{WUE}}_{\text{ET}}=\frac{\text{total drybiomass}}{\text{total amount of evapotranspiration}}$$

(6) $${\text{WUE}}_{\text{I}}=\frac{\text{total drybiomass}}{\text{total amount of irrigation}}$$

(7) $${\text{WUE}}_{\text{T}}=\frac{\text{total drybiomass}}{\text{total amount of transpiration}}$$

### Statistical analysis

All data were analyzed with SPSS software (version 13.0, SPSS Inc., Chicago, IL, USA). The data were firstly tested for normality and homogeneity using the Shapiro-Wilks test and the Cochran’s C-test, respectively. Then, differences between either irrigation regimes or soil clay content for the variables measured were tested using two-ways analysis of variance. When significant differences were detected, multiple comparisons of means were carried out with Duncan’s test at a 5% confidence level. In addition, a linear regression analysis was carried out to determine the relationship between (i) WUE and δ^13^C_organs_, (ii) WUE and δ^15^N_organs_, and (iii) δ^13^C_organs_ and δ^13^C_whole-plant_. Pearson correlation analysis was performed to test for correlations among δ^15^N_organs_, δ^13^C_organs_, [C] _organs_, C allocation rate to diverse organs, SPAD of flag leaves, and crack volume at a 5% confidence level.

## Results

### SPAD and panicle length

**Figure 1:**
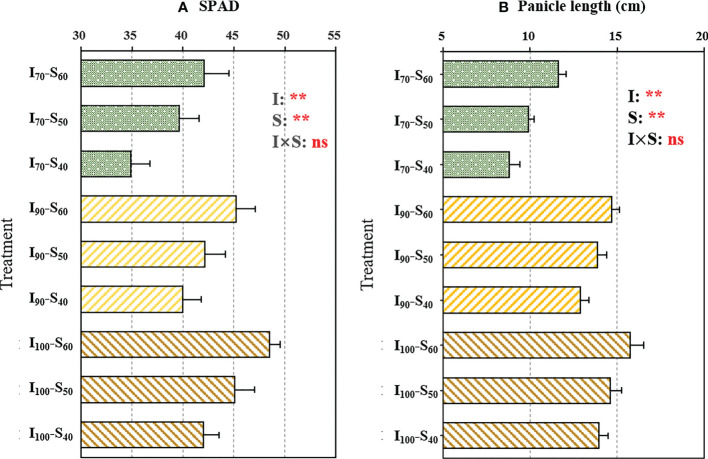
The output of two-way analysis of variance (ANOVA) for **(A)** SPAD readings of flag leaves and **(B)** panicle length as influenced by different water regimes and soil clay contents (mean ± SD; n = 4). I_70_, I_90_ and I_100_ represent irrigation regimes of flooding with 30mm (upper limit) as the soil water reaches 70% of saturation (lower limit); flooding with 30mm (upper limit) as the soil water reaches 90% of saturation (lower limit) and flooding with 30mm (upper limit) as the soil water reaches 100% of saturation (lower limit). S_40_, S_50_, and S_60_ indicate soil clay content with 40%, 50% and 60% respectively. I, S, and I×S indicate irrigation regime, soil type, and the interaction between irrigation regime (I) and soil type (S), respectively. ns and ** represent no significance and *p*<0.01, respectively.

There were significant differences (*p*< 0.01) in the SPAD values under different water regimes and soil clay contents (**Figure 1A**). The AWD application (I_90_ and I_70_) decreased the SPAD values compared with flooding regime, whereas the elevated clay content significantly increased the SPAD. Compared to I_100_, the SPAD values under the I_90_ and I_70_ regimes decreased by 6.07% and 14.01%, respectively, across soil clay contents. The SPAD values under S_50_ and S_60_ increased by 8.42% and 16.08%, respectively, compared to S_40_, across water regimes. There was no significant (*p* > 0.05) interaction between the water regime and clay content on SPAD values. In addition, as shown in **Figure 1B**, the panicle length was significantly influenced by irrigation as well as soil clay content. Across soil clay content, the I_70_ and I_90_ regimes significantly decreased the panicle length by 31.60% and 6.49%, respectively, compared to I_100_. Across irrigation regimes, the panicle length notably increased with the increased clay content (*p*<0.01).

### Biomass, ET, and WUE

**Figure 2:**
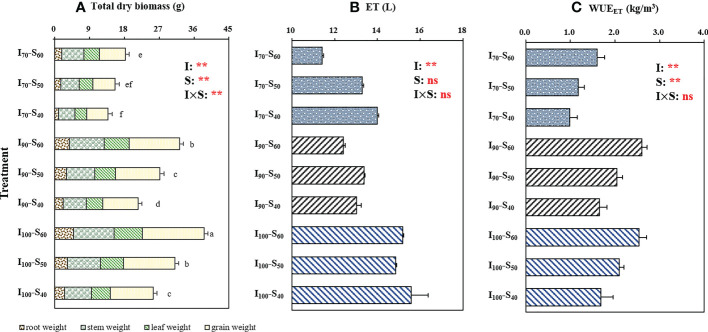
The effect of treatment and output of two-way analysis of variance (ANOVA) for **(A)** dry biomass of grain, stem, leaf, and root, **(B)**evapotranspiration (ET), and **(C)** WUE_ET_ of rice as influenced by different water regimes and soil clay contents (mean ± SD; n = 4). I_70_, I_90_ and I_100_ represent irrigation regimes of flooding with 30mm (upper limit) when the soil water reaches 70% of saturation (lower limit); flooding with 30mm (upper limit) as the soil water reaches 90% of saturation (lower limit); and flooding with 30mm (upper limit) as the soil water reaches 100% of saturation (lower limit). S_40_, S_50_, and S_60_ indicate soil clay content with 40%, 50% and 60% respectively. Different letters mean significant differences (*p*<0.05). I, S, and I×S indicate irrigation regime, soil type, and the interaction between irrigation regime (I) and soil type (S), respectively. ns and ** represent no significance and *p*<0.01, respectively..

**Figure 2** shows the effects of water regimes and soil clay contents on the dry biomass of rice organs, evapotranspiration (ET) and WUE_ET_. The two-way analysis of variance revealed significant (*p*< 0.01) differences between the total dry biomass of rice due to the application of different water regimes and soil clay contents (**Figure 2A**). The total biomass notably increased with elevating clay content, but decreased with a reduction in lower-limit of AWD. Compared to S_40_, the soil treatment S_50_ and S_60_ increased the total biomass by 21.72% and 46.65%, respectively, across irrigation regime. The ET was only significantly affected by the irrigation regimes (*p*<0.01). The ET under I_100_ was significantly higher than that under I_90_ and I_70_ regimes, across soil clay content. There were also significant (*p*< 0.01) differences for total transpiration under different water regimes and soil clay contents (**Supplementary Figure 2C**). With reduction in the lower-limit from 100% to 70% of saturated water content, the transpiration values decreased by 16.39% across soil clay contents. In addition, the transpiration value under S_40_ was 15.62% greater than that under S_60_, across water regimes. There was no significant interaction between the water regime and soil clay content on transpiration (*p* > 0.05).

**Figure 2C** showed significant (*p*< 0.01) differences in WUE_ET_ under different water regimes and soil clay contents. I_100_ resulted in the highest WUE_ET_ while the lowest WUE_ET_ value was observed under I_70_, when analyzed across the soil clay contents. With the increase in soil clay content, the WUE_ET_ significantly increased. WUE_ET_ under S_50_ and S_60_ increased by 22.97% and 56.08%, respectively, compared to S_40_, across the water regimes. As shown in **Figure 2C**, no interaction (*p* > 0.05) on WUE_ET_ was found for the water regime and soil clay content treatments. The effect of water regimes and soil clay contents on the WUE_T_ and WUE_I_ is shown in **Supplementary Figure 3**. Increased clay content significantly enhanced both WUE_T_ and WUE_I_. For WUE_T_, across soil clay contents, the highest value and the lowest value were was found in I_90_ and I_70_ application, respectively.

### C concentration and allocation

For all organs of rice, leaf C concentration ([C]) was significantly affected by soil clay content application (*p*<0.05, **Table 1**). The C allocation in the grain and leaf was both affected (*p*< 0.01) by water regimes and soil clay contents (**Table 1**). Differences (*p*< 0.01) in stem C allocation were also found under the different water regimes. The root C allocation varied with the soil clay contents (*p*< 0.05). Specifically, C allocation in the leaf was higher (*p*< 0.01) under I_70_ than that under I_100_, across the soil clay content treatments, whereas a contrary trend was observed for grain. The stem C allocation was highest in I_70_ and lowest in I_90_. With increased clay content, across the irrigation treatments, the C allocation in the grain markedly decreased, but the C allocation in the root significantly increased. For example, the highest value of root C allocation, as an average, was 11.72% under S_60_ while the lowest value was 9.30% in S_40_.

**Table 1:** The output of treatments and two-way analysis of variance (ANOVA) for the carbon concentration ([C]) values in grain, stem, leaf, and root, and C allocation of grain, stem, leaf, and root subjected to different water regimes and soil clay contents.

	Leaf C		Stem C		Root C		Grain C	
Treatment	Concentration (%)	Allocation (%)	Concentration (%)	Allocation (%)	Concentration (%)	Allocation (%)	Concentration (%)	Allocation (%)
I_100_-S_40_	33.92 ± 2.69ab	23.74 ± 1.66b	38.49 ± 1.17a	19.00 ± 0.54ab	37.94 ± 6.72a	9.91 ± 1.29ab	42.20 ± 0.20a	47.36 ± 1.2a
I_100_-S_50_	31.41 ± 2.74b	21.58 ± 2.38c	39.63 ± 0.35a	19.57 ± 0.04ab	43.97 ± 5.05a	11.80 ± 2.31a	42.67 ± 0.13a	47.06 ± 0.12a
I_100_-S_60_	35.42 ± 0.62a	24.33 ± 0.44b	41.51 ± 6.68a	19.83 ± 3.31ab	40.34 ± 13.2a	12.75 ± 3.81a	41.38 ± 1.04a	43.09 ± 2.5b
I_90_-S_40_	33.85 ± 1.35ab	25.16 ± 0.46b	34.08 ± 8.46a	17.55 ± 3.65b	39.18 ± 2.36a	10.41 ± 0.94ab	41.8 ± 1.59a	45.46 ± 2.95ab
I_90_-S_50_	35.41 ± 1.95a	24.48 ± 1.52b	38.25 ± 0.62a	19.17 ± 0.6ab	38.44 ± 3.83a	10.83 ± 1.01a	42.34 ± 0.52a	45.52 ± 1.57ab
I_90_-S_60_	35.47 ± 0.45a	25.33 ± 0.25b	38.16 ± 0.52a	19.16 ± 0.81ab	40.76 ± 5.65a	11.96 ± 1.60a	42.51 ± 0.75a	43.55 ± 0.99b
I_70_-S_40_	34.52 ± 1.91a	27.57 ± 1.21a	36.97 ± 2.24a	21.08 ± 1.13a	38.64 ± 3.81a	7.57 ± 1.06b	42.25 ± 0.96a	43.79 ± 2.96b
I_70_-S_50_	34.41 ± 1.89a	27.24 ± 0.80a	38.22 ± 0.40a	21.7 ± 0.46a	40.07 ± 7.22a	10.80 ± 2.09a	41.82 ± 0.53a	40.27 ± 2.17c
I_70_-S_60_	35.40 ± 0.50a	28.63 ± 0.61a	38.13 ± 0.94a	21.74 ± 0.98a	39.3 ± 7.71a	10.43 ± 1.19ab	41.97 ± 0.59a	39.2 ± 0.28c
F test I	ns	**	ns	**	ns	ns	ns	**
F test S	*	**	ns	ns	ns	*	ns	**
F *I×S*	ns	ns	ns	ns	ns	ns	ns	ns

Values are mean ± S.D. (n = 4) for each measurement. I_70_, I_90_ and I_100_ represent irrigation regimes of flooding with 30mm (upper limit) as the soil water reaches 70% of saturation (lower limit); flooding with 30mm (upper limit) as the soil water reaches 90% of saturation (lower limit) and flooding with 30mm (upper limit) as the soil water reaches 100% of saturation (lower limit). S_40_, S_50_, and S_60_ indicate soil clay content with 40%, 50% and 60% respectively. Different letters mean significant differences (p<0.05) according to the Duncan’s test. I, S, and I×S indicate irrigation regime, soil type, and the interaction between irrigation regime (I) and soil type (S), respectively. ns, *, ** and represent no significance, 0.01<p<0.05, and p<0.01, respectively.

Under the I_100_ and I_70_ regimes, significant differences were found among the three soil clay contents for C allocation of several organs. However, under the I_90_ regime, it was similar among the three soil clay contents for all organs. For instance, when increasing the soil clay content from 40% to 60%, the grain C allocation decreased by 9.03% in I_100_ and 10.48% in I_70_. The interaction between the water regimes and clay contents was not significant (*p* > 0.05) for any [C] and C allocation in the different organs.

### Variations of δ^13^C in plant organs and whole-plant

**Figure 3:**
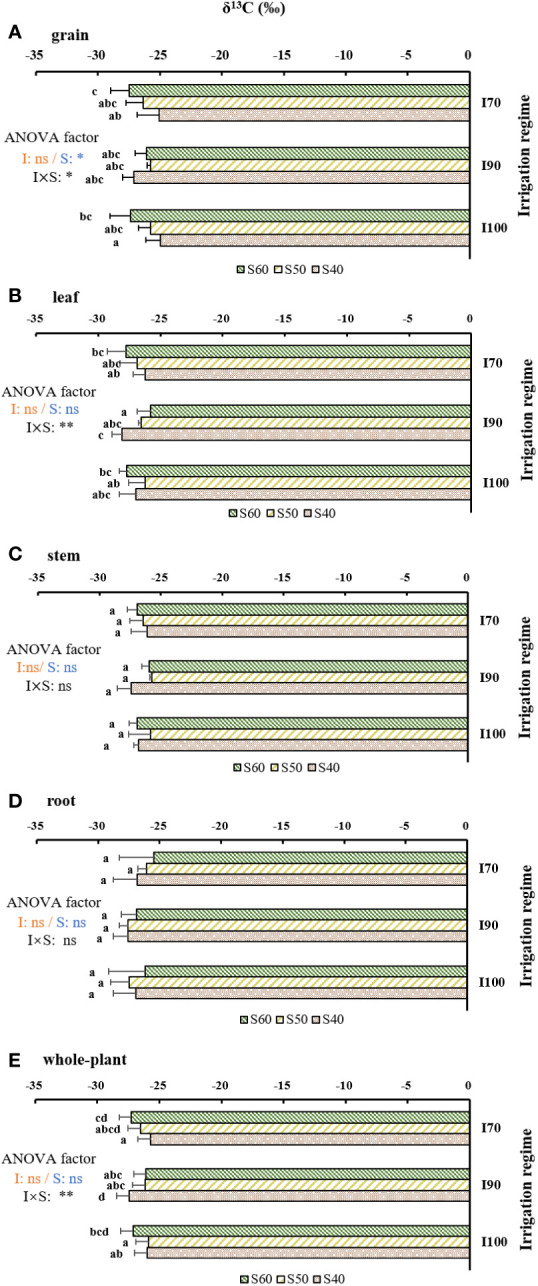
The effects of treatments and output of two-way analysis of variance (ANOVA) for δ^13^C values in **(A)** grain, **(B)** leaf, **(C)** stem, **(D)** root and **(E)** whole-plant as influenced by different water regimes and soil clay contents (mean ± SD; n = 4). I_70_, I_90_ and I_100_ represent irrigation regimes of flooding with 30mm (upper limit) as the soil water reaches 70% of saturation (lower limit); flooding with 30mm (upper limit) as the soil water reaches 90% of saturation (lower limit) and flooding with 30mm (upper limit) as the soil water reaches 100% of saturation (lower limit). S_40_, S_50_, and S_60_ indicate soil clay content with 40%, 50% and 60% respectively. Different letters mean significant differences (*p*<0.05). I, S, and I×S indicate irrigation regime, soil type, and the interaction between irrigation regime (I) and soil type (S), respectively. ns, *, ** and represent no significance, 0.01<*p*<0.05, and *p*<0.01, respectively.

The δ^13^C_grain_ was affected (*p*< 0.05) by the soil clay content and the interaction between water regime and soil clay content (**Figure 3A**). With the increasing soil clay content, the δ^13^C_grain_ decreased under I_70_ and I_100_ regime, but showed a different trend under the I_90_ regime. Across water regimes, the δ^13^C_grain_ under S_40_ (-25.70‰) was significantly higher than that under S_60_ (-26.97‰). The lowest and highest δ^13^C_grain_ (-27.49‰–24.94‰) were found under the I_70_-S_60_ and I_100_-S_40_ treatments, respectively.

The δ^13^C values in both leaf and the whole-plant level were significantly influenced by the interaction of water regimes and soil clay contents (**Figure 3B, E**, *p*<0.01). For I_70_, the δ^13^C_leaf_ was highest in S_40_, followed by S_50_ and S_60_. For I_90_, the δ^13^C_leaf_ showed an increasing trend with the elevated soil clay content. For I_100_, the δ^13^C_leaf_ decreased with clay content in the order of S_50_, S_40,_ and S_60_ applications. The δ^13^C_whole-plant_ showed similar variations as the δ^13^C_leaf_ with varied soil clay contents. The lowest δ^13^C_leaf_ and δ^13^C_whole-plant_ existed under I_90_-S_40_, while the lowest values of δ^13^C_leaf_ and the δ^13^C_whole-plant_ existed under the I_90_-S_60_ and I_70_-S_40_, respectively. In addition, δ^13^C increased slightly from shoot to root. Especially, under I_100_-S_40_, the δ^13^C in grain was significantly higher than that in other organs.

### Relationships between WUE and carbon isotopic composition, and nitrogen isotopic composition

**Figure 4:**
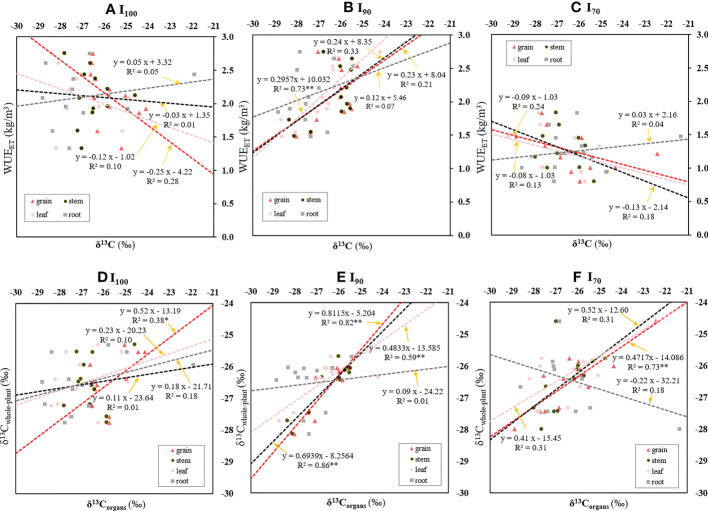
Relationships between WUE_ET_ and carbon isotope composition of diverse rice organs (δ^13^C_organs_) **(A–C)**; and between δ^13^C _whole-plant_ and δ^13^C_organs_ **(D–F)** as influenced by different water regimes. *, ** and represent 0.01<*p*<0.05, and *p*<0.01, respectively.

There was no significant relationship found between WUE_ET_ and *δ* ^13^C in rice organs based on the pooled data of nine treatments (**Supplementary Figure 4**). Nevertheless, across the soil clay content treatments, there was a significantly positive relationship between WUE_ET_ and δ^13^C_leaf_ (R^2^=0.73, *p*<0.01) under I_90_ irrigation regime (**Figure 4B**), whereas there was no significant relationship between *δ* ^13^C_organs_ and WUE_ET_ under I_100_ or I_70_ AWD regimes (**Figure 4A, C**). The WUE_I_ and WUE_T_ presented a similar trend for their relationships with rice *δ* ^13^C_organs_ (**Supplementary Figure 5**). Significant relationships were found between (i) WUE_I_ and δ^13^C_leaf_ (R^2^=0.78, *p*<0.01), (ii) WUE_I_ and δ^13^C_stem_ (R^2^=0.39, *p*<0.05), and (iii) WUE_T_ and δ^13^C_leaf_ (R^2^=0.71, *p*<0.01) under I_90_ regime. Across the irrigation regimes, no significant relationship was observed between δ^13^C_organs_ and WUE_s_ under any soil treatments (*p*>0.05, data not shown).

Furthermore, a linear regression was carried out to reveal the variation tendency of the δ^13^C_whole-plant_ with the δ^13^C_organs_ under three water regimes (**Figure 4D-F**). The δ^13^C_whole-plant_ could be expressed as a function of the δ^13^C_grain_ under I_100_ (R^2^=0.38, *p*< 0.05) and I_70_ regimes (R^2^=0.73, *p*< 0.01), respectively. For I_90_ regime, there were significantly positive relationships between (i) δ^13^C_whole-plant_ and δ^13^C_grain_ (R^2^=0.82, *p*<0.01), (ii) δ^13^C _whole-plant_ and δ^13^C_stem_ (R^2^=0.86, *p*<0.01), and (iii) δ^13^C_whole-plant_ and δ^13^C_leaf_ (R^2^=0.59, *p*<0.01).

**Figure 5:**
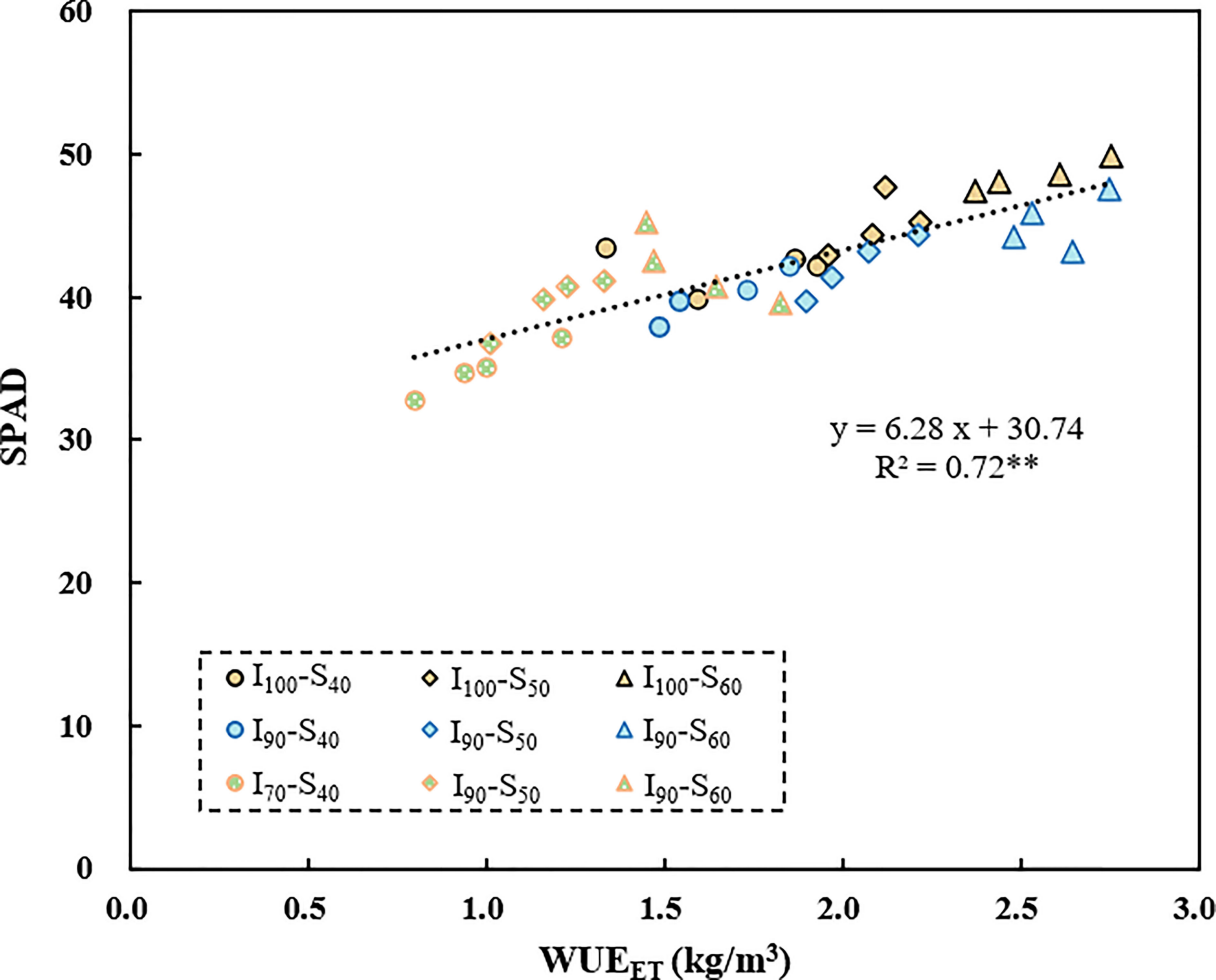
The relationship between SPAD and WUE_ET_ as influenced by different water regimes and soil clay contents. ** represents *p*<0.01.

**Figure 6:**
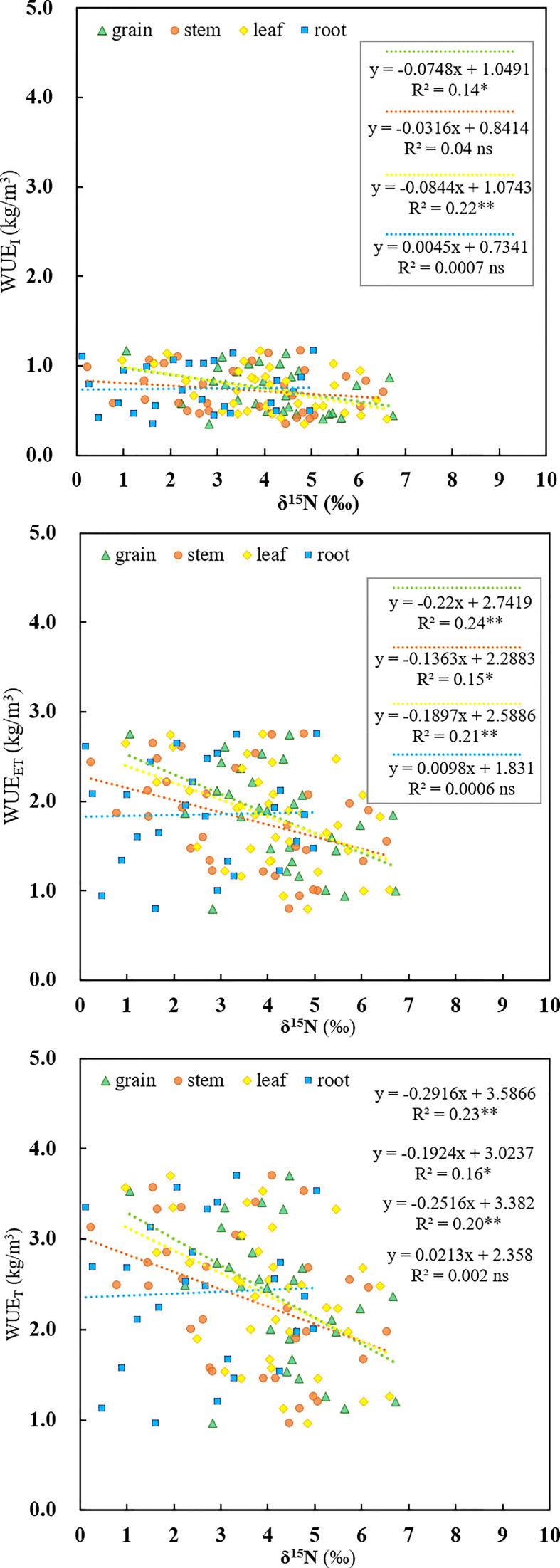
Relationships between three kinds of water use efficiency (WUE_I_, WUE_ET_, WUE_T_) and nitrogen isotope composition of diverse rice organs (δ^15^N_grain_, δ^15^N_stem_, δ^15^N_leaf_, δ^15^N _root_) as influenced by different water regimes and soil clay contents. ns, *, ** and represent no significance, 0.01<*p*<0.05, and *p*<0.01, respectively.

The SPAD values are positively correlated with the WUE_ET_ across the irrigation regimes (R^2^=0.72, *p*<0.01, **Figure 5**). Additionally, there was a significantly negative correlation between WUE_I_ and δ^15^N_grain_ (R^2^=0.14, *p*<0.05), and between WUE_I_ and δ^15^N_leaf_ (R^2^=0.22, *p*<0.01) (**Figure 6**) based on pooled data. The WUE_ET_ was negatively correlated with δ^15^N in grain (R^2^=0.24, *p*<0.01), stem (R^2^=0.15, *p*<0.05), and leaf (R^2^=0.21, *p*<0.01) (**Figure 6**). Similarly, the WUE_T_ was significantly and negatively correlated with grain (R^2^=0.23, *p*<0.01), stem (R^2^=0.16, *p*<0.05), and leaf (R^2^=0.20, *p*<0.01) (**Figure 6**).

### Pearson correlation analysis

Pearson’s correlations among SPAD, δ^13^C and δ^15^N of diverse organs are shown in **Table 2**. The δ^15^N_grain_ (r= -0.50, *p<* 0.01), δ^15^N_stem_ (r= -0.42, *p<* 0.05) and δ^15^N_leaf_ (r= -0.44, *p<* 0.01) showed a strong negative correlation with SPAD value. In addition, a significantly positive relation between (i) *δ* ^13^C_grain_ and *δ* ^13^C_stem_ (r= 0.40, *p*<0.05), (ii) *δ* ^13^C_grain_ and *δ* ^13^C_leaf_ (r= 0.41, *p*<0.05), and (iii) *δ* ^13^C_stem_ and *δ* ^13^C_leaf_ (r= 0.54, *p*<0.01) were found in the current study.

**Table 2:** Pearson correlation coefficients for SPAD reading, δ^13^C values in grain, stem, leaf, and root, and δ^15^N of grain, stem, leaf, and root.

	SPAD	δ^13^C_grain_	δ^13^C_stem_	δ^13^C_leaf_	δ^13^C _root_	δ^15^N_grain_	δ^15^N_stem_	δ^15^N_leaf_	δ^15^N _root_
SPAD	1	-0.09	0.00	0.01	0.07	-0.50**	-0.42*	-0.44**	0.114
δ^13^C_grain_		1	0.40*	0.41*	-0.27	0.11	-0.02	0.12	-0.18
δ^13^C_stem_			1	0.54**	-0.02	-0.02	-0.05	-0.12	-0.16
δ^13^C_leaf_				1	-0.23	0.03	0.01	-0.14	-0.30
δ^13^C _root_					1	-0.23	-0.30	-0.11	0.29
δ^15^N_grain_						1	0.48*	0.30	0.13
δ^15^N_stem_							1	0.29	0.22
δ^15^N_leaf_								1	-0.07
δ^15^N _root_									1

*indicates significant differences at 0.01<p<0.05; **indicates significant differences at p<0.01.

The correlations between the crack volume, [C] and the C allocation of diverse organs are shown in **Supplementary Table 1**. With increasing crack volume, the C allocation to grain decreased in varying degrees (r=-0.69, *p*<0.01) while the C allocation to stem and leaf increased (r=0.47, *p*<0.01 and r=0.81, *p*<0.01, respectively). However, the crack volume did not have a significant correlation with any organs [C] (**Table S1**). Furthermore, the [C]_grain_ was negatively correlated with the [C]_stem_ (r=-0.40, *p*< 0.05). For the C allocation of diverse organs, the C allocation to stem and leaf in rice showed a strong negative correlation with grain (r=-0.66, *p*<0.01 and r=-0.69, *p*<0.01, respectively).

## Discussion

### Effects of irrigation regimes and soil clay contents on WUE_s_

Water-saving irrigation regime, such as AWD, can improve WUE while maintaining or even increasing rice yield (Song et al., 2021). Consistent with this, in the current study, an increased WUE_T_ of rice was observed when reducing the lower-limit of irrigation from 100% to 90% of saturated water content, though the differences were not statistically significant (**Supplementary Figure 3**). Nevertheless, when the irrigation lower-limit further decreased to 70% of saturated water content, a significantly lower WUE_T_ was observed compared to I_100_ and I_90_ regimes (**Supplementary Figure 3**), which mainly due to the higher degree of water deficit in AWD under the I_70_ treatment (Carrijo et al., 2017). It has been reported that slight drought stress could induce partially stomatal closure, hereby decreasing transpiration and improving WUE_T_ (Ma et al., 2021). Whereas, severe drought stress impaired carbon fixation and physiological disorders in plants, such as the reduction of photosynthetic capacity, leading to decreased WUE_T_ (Wang et al., 2020). A previous study also indicated that SPAD values of leaves were closely related to photosynthetic capacity (Wang et al., 2012). The significantly lower SPAD values of I_70_ compared to I_90_ and I_100_ (**Figure 1A**) could indicate the decreased photosynthetic capacity in I_70_. Consequently, the biomass of I_70_ was significantly decreased with a reduction in WUE_T_ compared to I_100_ (**Figure 2A** and **Supplementary Figure 3**). Similarly, WUE_ET_ of I_70_ was observed significantly lower than those of I_100_ and I_90_ (**Figure 2C**).

In addition to irrigation regimes, the clay content of soil also affected the WUE_ET_ and WUE_T_ of rice. In the present study, the WUE_ET_ and WUE_T_ increased with the elevation of soil clay contents (**Figure 2C** and **Supplementary Figure 3**) (Fotovat et al., 2007). Similarly, Dou et al. (2016) found that, compared to sand soils, the plants grown in clay soil exhibited higher WUE due to better nitrogen status by increased availability of organic matters and water in clay soil as well as improved nitrogen uptake for plants. Consistent with this, higher SPAD values was observed in the presence of elevated clay content (**Figure 1A**), which could increase plant photosynthesis capacity (Wang et al., 2012), in line with higher biomass production under this treatment (**Figure 2A**). WUE_I_ showed a similar changing trend under different irrigation regimes with varied clay content as WUE_ET_ and WUE_T_ (**Figure 2C** and **Supplementary Figure 3**), implying that the three WUEs were mainly regulated by photosynthesis associated with biomass production (Fotovat et al., 2007).

### Effect of irrigation regimes and soil clay contents on carbon allocation and δ^13^C values of different organs

Previous studies reported that carbon allocation in different organs of crops could be induced by irrigation regimes (Arndt and Wanek, 2002; Oliver et al., 2019; Liu et al., 2020). In this study, there were significant differences in C allocation to different organs of rice grown under different irrigation regimes (**Table 1**). Regarding aboveground organs, C allocation to grain decreased significantly with reduced level of irrigation, but a contrary trend was observed for stem and leaf (**Table 1**). This result is in contrast to the finding of Song et al. (2021) showing that AWD promoted carbohydrate transfer from stem to grain compared to flooding irrigation. A possible explanation is that moderate drought stress could increase the transfer of C to grain, but this could not occur under severe drought stress due to the drought damage (Oliver et al., 2019). Additionally, Liu et al. (2020) suggested that when plants were exposed to drought stress, the new carbohydrates were preferentially transported from shoot to root, and consequently resulted in an increase of C allocation to root. However, in the current study, the C allocation to belowground organs were not affected by irrigation regimes (**Table 1**). The phenomenon may be attributed to the larger cracks formed in AWD, which could stretch and tear the roots and might influence the C allocation among organs by inducing root-pruning signal (**Supplementary Table 1**) (Bordoloi et al., 2020).

In addition to irrigation regime, soil clay content also affected the C allocation of different organs in rice (**Table 1**). Under I_100_ and I_70_ treatment, C allocation to grain varied with clay contents (**Table 1**), possibly due to the changed water and nitrogen status of soil as affected by the increased clay contents (Fotovat et al., 2007), as previous studies indicated that C allocation of plants differed significantly with respect to the water and fertilizer conditions (Xu et al., 2007). Nonetheless, under the I_90_ treatment, similar C allocation was observed among organs under different clay contents (**Table 1**). The possible reason for this discrepancy is ascribed to the soil clay content-induced cracks. For the I_100_ regime, no soil crack was observed with high soil water potential (**Supplementary Figure 1**). Therefore, the different clay contents under the same irrigation regime led to the differences in the ability of soils to retain C, water, and nutrient ions, which might impact the C allocation to grain and leaf (**Table 1**) by affecting plant photosynthetic capacity (Zhao et al., 2021). For I_70_, the crack volumes were positively correlated with soil clay contents (**Supplementary Figure 1**), which in turn may significantly influence the soil water and fertilizer contents due to enhanced preferential flow (Cheng et al., 2021). Hence, the C allocation pattern in rice was changed accordingly (**Table 1**). Regarding I_90_, the potential increase in N leaching loss associated with enlarged crack volumes (detailed information shown in Wang et al., 2022) was largely offset by the rise in nitrogen retention capacity associated with increased clay content, thereby restricting the variation in the availability of water and nitrogen in soil and C allocation in rice.

It has been widely accepted that variation in allocation patterns in plants could result in δ^13^C changes in plant organs (De Souza et al., 2005). Compared to autotrophic organs (leaves) that supply plants with carbon, heterotrophic organs (stems, grains and roots) tend to be rich in ^13^C (Zhang et al., 2015). The difference of δ^13^C values among organs under I_90_ was significantly smaller than those under I_100_ and I_70_, though there was still a tendency for increased δ^13^C from leaf to root (**Figure 3**). Other potential reasons for organ-specific differences in δ^13^C could be related to the differences in fractionation processes during the enzymatic reactions, and the chemical composition of different organs, such as the amounts of lipids and lignin (Kano-nakata et al., 2014; Zhang et al., 2015). It was found that δ^13^C_whole-plant_ also showed a strong correlation with δ^13^C_leaf_ under the I_90_ regime (**Figure 4E**), which was in agreement with the finding of Gouveia et al. (2019b). Moreover, δ^13^C_grain_ showed the most consistent and significant correlation with δ^13^C _whole-plant_ under three irrigation regimes (I_100_, I_90_ and I_70_) (**Figure 4D-F**). This result may be attributed to the isotopic fractionation during the allocation and transfer processes of carbon within plants (Sanchez-Bragado et al., 2014). As shown in equation 4, δ^13^C _whole-plant_ was the integrated δ^13^C values of different organs. Similarly, as indicated by Araus et al. (1993) and Zhu et al. (2021), δ^13^C_grain_ was the result of the combined δ^13^C values of assimilates produced by different photosynthetic organs, such as the ears and the flag leaves, responsible for grain filling after anthesis, and the remobilization of nonstructural carbohydrate reserves stored in the specific organ, such as the sheaths and culm. Thus, accordingly, δ^13^C_grain_ showed the most consistent correlation with δ^13^C _whole-plant_ across different treatments, and might be a priority indicator of δ^13^C _whole-plant_. The strong negative correlation between [C]_grain_ and [C]_stem_ found in this study (*p*<0.05, r=-0.40, **Supplementary Table 1**) further supported the aforementioned speculation. However, there were weak correlations between other δ^13^C_organs_ (δ^13^C_leaf_, δ^13^C_stem_, δ^13^C_root_) and δ^13^C_whole-plant_ under I_100_ or I_70_ treatment (**Figures 4D, F**). We speculated that this weak correlation may be related to variations in carbon allocation patterns under the two regimes with varied soil clay contents (**Table 1**).

### Relationship between δ^13^C_organs_ and WUE and δ^13^C_whole- plant_

Farquhar and Richards (1984) reported that C_i_/C_a_ was negatively related to WUE_i_, while C_i_/C_a_ was negatively related to organ δ^13^C. Thus, it could be concluded that there was a positive relationship between organ δ^13^C and WUE_i_. However, in this study, there was no significant correlation between δ^13^C_organs_ and WUE_whole-plant_ based on pooled data (**Supplementary Figure 4**). This phenomenon could be explained as follows. First, the possible varied leaf boundary layer conductance among different treatments might result in WUE_i_ independence from stomatal conductance, C_i_/C_a_ and δ^13^C (Cernusak et al., 2009). In this study, although we did not measure leaf boundary layer conductance between the intercellular spaces and the atmosphere, the significantly higher water loss through transpiration as well as the increased leaf biomass for I_100_, compared to I_70_ (**Figure 2A** and **Supplementary Figure 2**), might indirectly indicate the varied microclimate and plant statuses caused by different irrigation regimes. Second, the degree of dark respiration, changed mesophyll conductance (g_m_) under different environmental conditions, and varied proportions of uncontrolled water loss to the transpiration might disturb the relationship between C_i_/C_a_, δ^13^C and A_n_/T (photosynthesis rate/transpiration rate) (Farquhar et al., 1989), which consequently result in a poor relationship between organs δ^13^C and WUE_whole-plant_. Interestingly, when the data were grouped into different irrigation regimes, relationships between δ^13^C and WUE_whole-plant_ varied with changed irrigation regimes (**Figure 4** and **Supplementary Figure 5**). For I_90_, there was a significant positive relationship between δ^13^C and WUE_whole-plant_, which was in agreement with the findings by Mininni et al. (2022). However, there were no significant relationships between the WUE and δ^13^C_whole-plant_ in I_100_ and I_70_ treatment. For I_100_, the poor relationship between δ^13^C of plant organs and WUE_whole-plant_ might be related to the relatively higher rate of panicle water loss. As indicated by Scartazza et al. (1998), most water loss through panicle was cuticular, which was independent of stomatal regulation and photosynthesis. Hence, high rates of panicle transpiration might disturb the relationships between δ^13^C and WUE in rice. In this study, increased panicle length (**Figure 1B**) as well as greater grain weight (**Figure 2A**) for I_100_ compared to I_90_ and I_70_ might indicate that the relatively higher proportions of panicle water loss to total transpiration would disturb the relationship between δ^13^C and WUE in rice under I_100_. Furthermore, changes of carbon allocation pattern for I_100_ with varied soil clay content (**Table 1**) might also lead to a breakdown in the relationship between δ^13^C_leaf_ and WUE_whole-plant_ (Wen et al., 2022). For I_70_, the weak relationship between δ^13^C and WUE_whole-plant_ (**Figure 4** and **Supplementary Figure 5**) might be due to physiological damages under severe water deficit, such as severely reduced photosynthetic enzyme activity and consequently disrupted photosynthetic process (Bogati and Walczak, 2022). In addition, for I_70_, the bigger cracks formed in this water-limited irrigation regime were associated with the stimulated leaching of water and nitrogen. Meanwhile, the tearing effect of cracks on the root system under the high clay content together with the reduced availability of water and nitrogen in I_70_ might deteriorate the physiological disorder process, which consequently results in breakdown of the relationship between δ^13^C_leaf_ and the WUE_whole-plant_.

### Relationship between organs δ^15^N and WUE

In contrast to the relationship between δ^13^C and WUE_whole-plant_, which varies with irrigation regimes, we found a significant negative correlation between δ^15^N_leaf_ and WUE_whole-plant_ based on pooled data (**Figure 6**). This result is consistent with the findings of Topbjerg et al. (2014) and Yousfi et al. (2012) but contrasting to the results of Cao et al. (2014). WUE_whole-plant_ is known to be highly associated with WUE_i_, controlled by either A_n_ or g_s_, or a combination of both, and could be improved by enhancing the A_n_ or by lowering g_s_. In this study, with the elevated soil clay content, the WUE_ET_ significantly increased (**Figure 2C**), mainly due to the increased photosynthetic capacity. SPAD has been extensively used to indicate the [N] and photosynthetic capacity in the leaf in the last few years, and a higher SPAD would mean a higher A_n_ (Fotovat et al., 2007). With the increased clay content, the [N]_leaf_ increased accordingly, thereby increasing the A_n_ and WUE_ET_. Consistent with this, SPAD was significantly positively correlated with WUE_ET_ in this experiment (**Figure 5**). In addition, if the increased WUE_i_ was only linked to SPAD, then a positive correlation between SPAD and δ^13^C_leaf_ associated with time-integrated WUE_i_ would be expected. However, in the present study, there was no clear relationship between SPAD and δ^13^C (*p*>0.05, **Table 2**), indicating that other factors may control increased WUE_ET_, such as decreased stomatal conductance in response to abiotic stress (Desrochers et al., 2022). As mentioned previously, SPAD had a significant positive correlation with clay content (**Figure 1A**). Under water-saving irrigation, with the increase of clay content, larger cracks formed in field could stretch and tear the roots, which is similar to the effect of root pruning (Bordoloi et al., 2020), which could decrease both stomatal conductance and transpiration due to the pruning-induced root signals (Ma et al., 2008; Feng et al., 2022). In this study, the large cracks observed under the same AWD regime with high clay content might suggest a strong stress signal generated by root pruning. Consequently, the stomatal conductance of rice was speculated to be reduced accordingly. Meanwhile, previous studies have shown that reduced g_s_ would lead to a reduction in the loss of ammonia and nitrous oxide, hence decreasing δ^15^N in leaf (Yousfi et al., 2012). Therefore, increased WUE_ET_ and WUE_T_ caused by reduced g_s_ (Yan et al., 2020) was expected to be negatively correlated with δ^15^N_leaf_, which is consistent with the results of this study.

Another reason for the negative relationship between ^15^N and WUE_whole-plant_ might be the decreased $\text{N}{\text{O}}_{3}^{-}$ transport from root to shoot of plants exposed to stress-condition (Yousfi et al., 2012). Due to the fractionation induced by nitrate reductase (NR), $\text{N}{\text{O}}_{3}^{-}$ not assimilated in the roots would be enriched in ^15^N and exported to shoots for assimilation, causing an increased δ^15^N in shoots relative to roots (Zhang et al., 2017). Hence, stress conditions would restrict $\text{N}{\text{O}}_{3}^{-}$ transport from the roots to the shoots, therefore increasing ^15^N in root while decreasing it in the shoots compared with the flooding irrigation (Yousfi et al., 2012). In our study, the clay contents in soil were positively correlated with SPAD (**Figure 1A**). Moreover, as shown in **Supplementary Figure 1**, the soil clay contents were also positively correlated with crack volume under AWD. As suggested aforementioned, higher crack volumes under AWD might imply a strengthened abiotic stress for crop, and consequently reduced $\text{N}{\text{O}}_{3}^{-}$ from root to shoot, and ultimately resulted in the decrease of δ^15^N in shoot. Thus, a negative relationship between SPAD and shoot δ^15^N was likely to be found (**Table 2**). In this study, the C and N allocation to roots were significantly increased with the elevated soil clay content (**Table 1**), which further demonstrated that the large crack reduced the export of $\text{N}{\text{O}}_{3}^{-}$ from roots to shoots. Moreover, the WUE_ET_ showed a significant positive correlation with SPAD (**Figure 5**), but a negative correlation with δ^15^N (**Figure 6**). Hence, the SPAD values tend to be negatively correlated with the δ^15^N (**Table 2**) and further supported that the increased WUE was probably due to a combination of A_n_ and g_s_. However, it should be noted that ^15^N in plants can also be influenced by the variations of soil nutrients (Tang et al., 2017), which was limited by the occurrence of cracks. In this case, the discrimination process for ^15^N during the N uptake tends to be slight, resulting in an increased ^15^N in plants while the WUE was decreased by the crack. Therefore, a significant negative relationship between the WUE and δ^15^N was found (**Figure 6**), but further trials are needed to examine the actual underlying mechanism.

## Conclusions

WUE_whole-plant_ generally increased with higher soil clay content. Variations in WUE_whole-plant_ were not consistent with variations in δ^13^C of organs under varied irrigation regimes with high clay contents. The rice δ^13^C _leaf_ was closely and positively related to the WUE_whole-plant_ under I_90_ regimes, whereas δ^13^C _organs_ was not related to WUE_whole-plant_ under I_100_ or I_70_ water regime. Among the organs, significant correlations were observed between *δ* ^13^C_grain_ and *δ* ^13^C_whole-plant_ under I_100_, I_90_ and I_70_ regimes. In addition, based on pooled data, WUE_whole-plant_ showed a significant negative correlation with δ^15^N_shoot_. Therefore, it is suggested that δ^13^C could not be used as a reliable indicator of differences in WUE_whole-plant_ associated with changes in irrigation regimes and clay content, whereas δ^15^N could be considered as an effective indicator of WUE_whole-plant_.

## Data availability statement

The original contributions presented in the study are included in the article/**Supplementary Material**. Further inquiries can be directed to the corresponding authors.

## Author contributions

ZCW and YAH designed the experiments. ZCW and JJL wrote the article and made critical revisions. CH and MHT helped in analyzing the data. YAH and RJQ supervised the experiments. RJQ, YSW, EA, HS, XPG edited and complemented the manuscript writing and its discussion. All authors contributed to the article and approved the submitted version.

## Funding

This study was funded by the Fundamental Research Funds for the Central Universities (B210202118), the National Natural Science Foundation of China (52079041,52179036). EA acknowledges multi-year support from The Startup Foundation for Introducing Talent of Nanjing University of Information Science & Technology (NUIST), Nanjing, China (Grant No. 003080) and the Jiangsu Distinguished Professor program of the People’s Government of Jiangsu Province.

## Conflict of interest

The authors declare that the research was conducted in the absence of any commercial or financial relationships that could be construed as a potential conflict of interest.

## Publisher’s note

All claims expressed in this article are solely those of the authors and do not necessarily represent those of their affiliated organizations, or those of the publisher, the editors and the reviewers. Any product that may be evaluated in this article, or claim that may be made by its manufacturer, is not guaranteed or endorsed by the publisher.
