# Supplementary material for: Relationships between stable isotope natural abundances (δ13C and δ15N) and water use efficiency in rice under alternate wetting and drying irrigation in soils with high clay contents
Source: Front Plant Sci. 2022 Dec 2;13:1077152. doi: 10.3389/fpls.2022.1077152 (PMC9756853; doi:10.3389/fpls.2022.1077152)
Supplement: Supplementary file 1 [file DataSheet_1.pdf]

Supplementary Material

1 Supplementary Information

1.1 Evaporation measurement

The surface evaporation loss under three clay contents was estimated by the water evaporated from containers maintained without plants ("bare" containers) for I_{100}, I_{90}, and I_{70} regimes. In the current experiment, the diameter of the pot with rice was only 16cm, and the canopy coverage gradually increased with the growth of rice. Therefore, the surface evaporation after late tillering stage significantly decreased and only accounted for a small proportion of evapotranspiration (Liu et al. 2016). Considering the fact mentioned above, the data of surface evaporation loss after late tillering stage was ignored in the calculation in this study.

Xiaoyin, L.; Guanyi, W.; Shihong, Y.; Junzeng, X.; Yijiang, W. Influence Factors and Characteristics of Transpiration and Evaporation in Water-Saving Irrigation Paddy Field under Different Temporal Scales. Nongye Jixie Xuebao/Transactions Chinese Soc. Agric. Mach. 2016, 47.

2 Supplementary Figures and Tables

2.1 Supplementary Figures

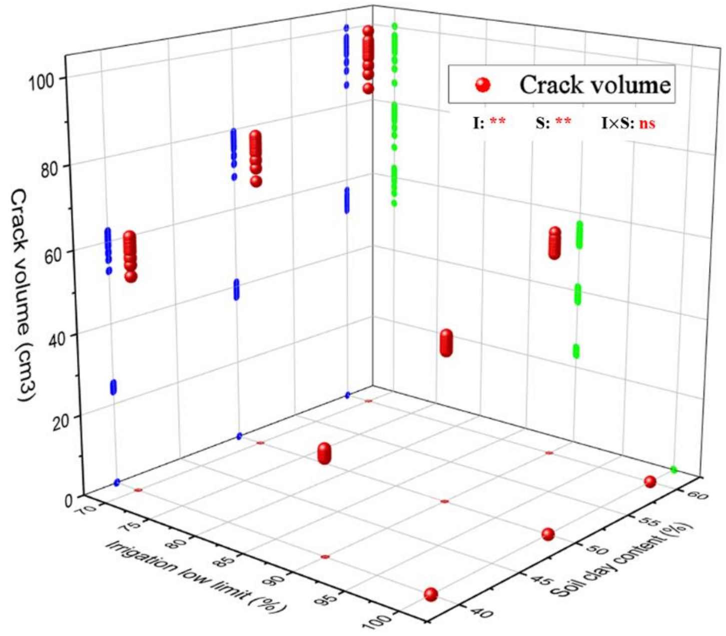

Supplementary Figure 1. The output of two-way analysis of variance (ANOVA) for Crack volumes as influenced by different water regimes and soil clay contents. I, S, and I×S indicate irrigation regime, soil type, and the interaction between irrigation regime (I) and soil type (S), respectively. ns, *, ** and represent no significance, 0.01<p <0.05, and p<0.01, respectively.

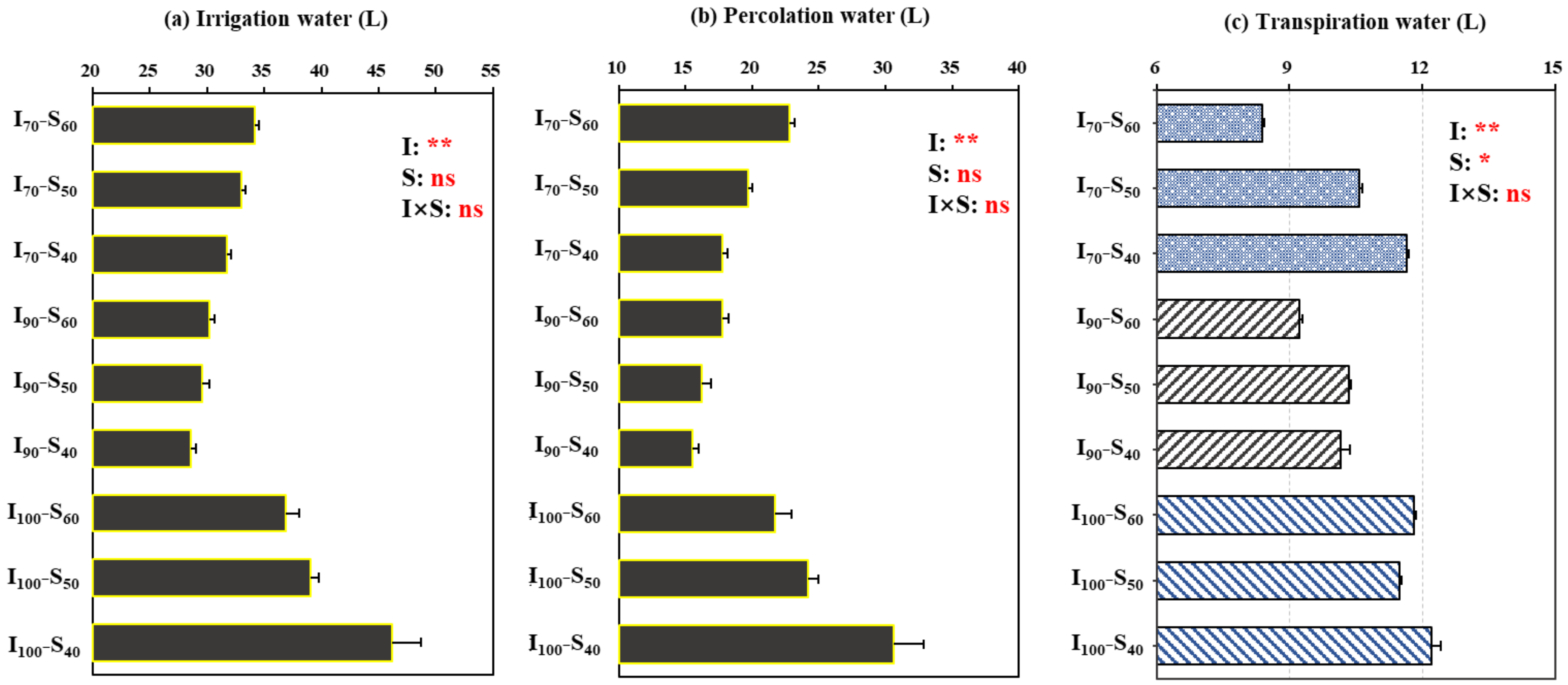

Supplementary Fig. 2 The output of two-way analysis of variance (ANOVA) for irrigation water and evapotranspiration as influenced by different water regimes and soil clay contents (mean ± SD; n = 4). I_{70}, I_{90} and I_{100} represent irrigation regimes of flooding with 30mm (upper limit) as the soil water reaches 70% of saturation (lower limit); flooding with 30mm (upper limit) as the soil water reaches 90% of saturation (lower limit) and flooding with 30mm (upper limit) as the soil water reaches 100% of saturation (lower limit). S_{40}, S_{50}, and S_{60} indicate soil clay content with 40%, 50% and 60% respectively. I, S, and I×S indicate irrigation regime, soil type, and the interaction between irrigation regime (I) and soil type (S), respectively. ns, *, ** and represent no significance, 0.01<p <0.05, and p<0.01, respectively

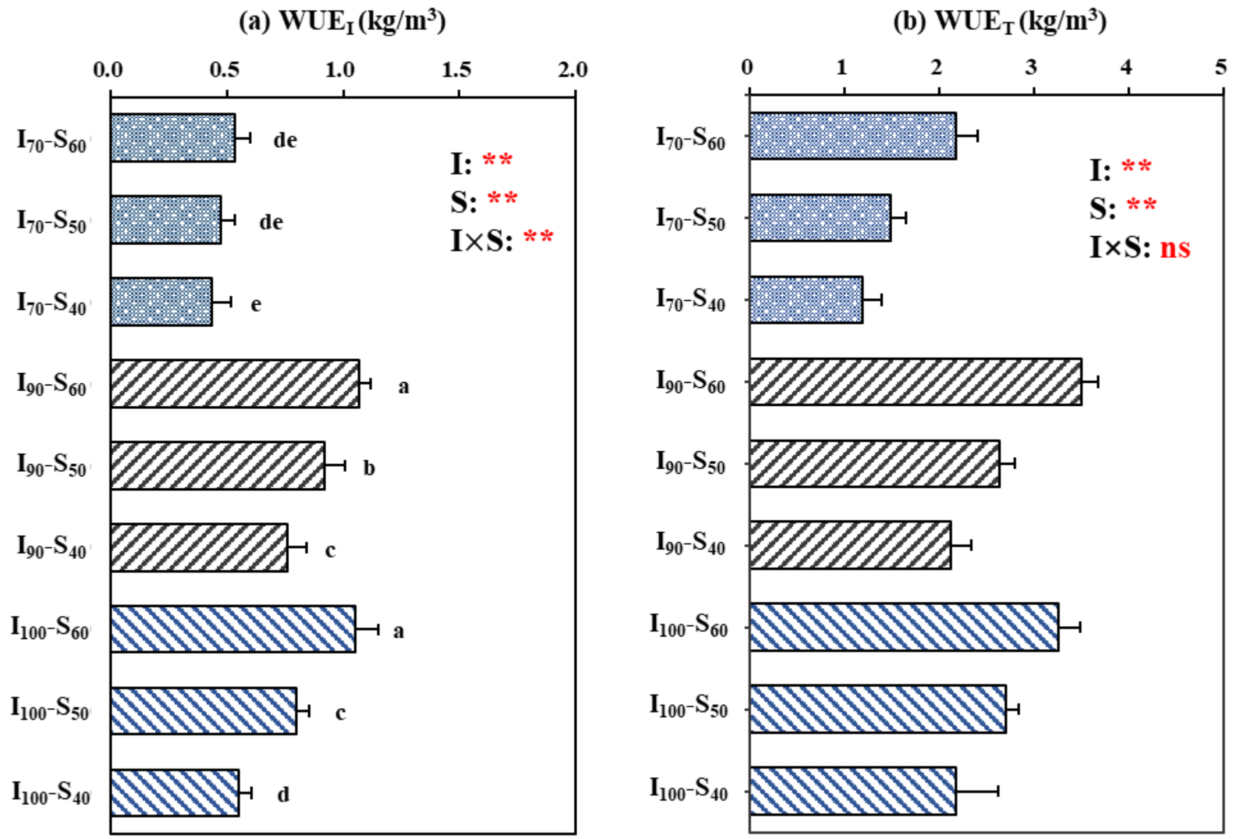

Supplementary Fig. 3 The effects of treatments and output of two-way analysis of variance (ANOVA) for (a) WUE_I and (b) WUE_T as influenced by different water regimes and soil clay contents (mean ± SD; n = 4). I_{70}, I_{90} and I_{100} represent irrigation regimes of flooding with 30mm (upper limit) as the soil water reaches 70% of saturation (lower limit); flooding with 30mm (upper limit) as the soil water reaches 90% of saturation (lower limit) and flooding with 30mm (upper limit) as the soil water reaches 100% of saturation (lower limit). S_{40}, S_{50}, and S_{60} indicate soil clay content with 40%, 50% and 60% respectively. Different letters mean significant differences (p<0.05). I, S, and I×S indicate irrigation regime, soil type, and the interaction between irrigation regime (I) and soil type (S), respectively. ns, *, ** and represent no significance, 0.01<p <0.05, and p<0.01, respectively.

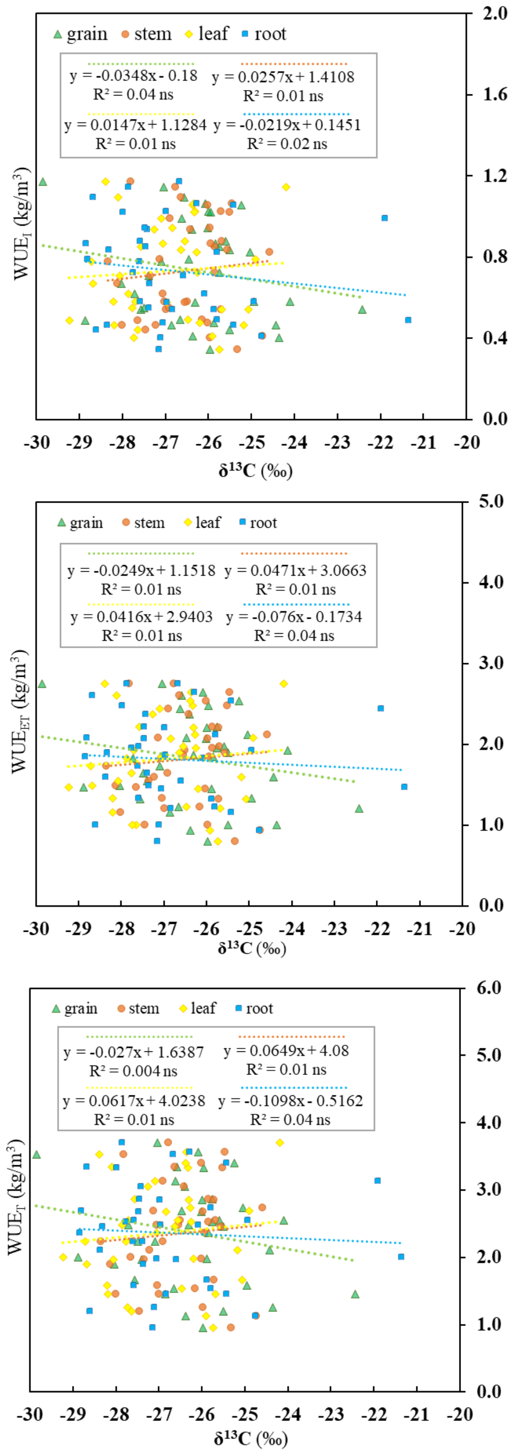

Supplementary Fig. 4 Relationship between carbon isotope composition of diverse rice organs ($\delta^{13}C_{grain}$, $\delta^{13}C_{stem}$, $\delta^{13}C_{leaf}$, $\delta^{13}C_{root}$) and three kinds of water use efficiency (WUE_I, WUE_{ET}, WUE_T) as influenced by water regimes and soil clay contents. ns represents no significance.

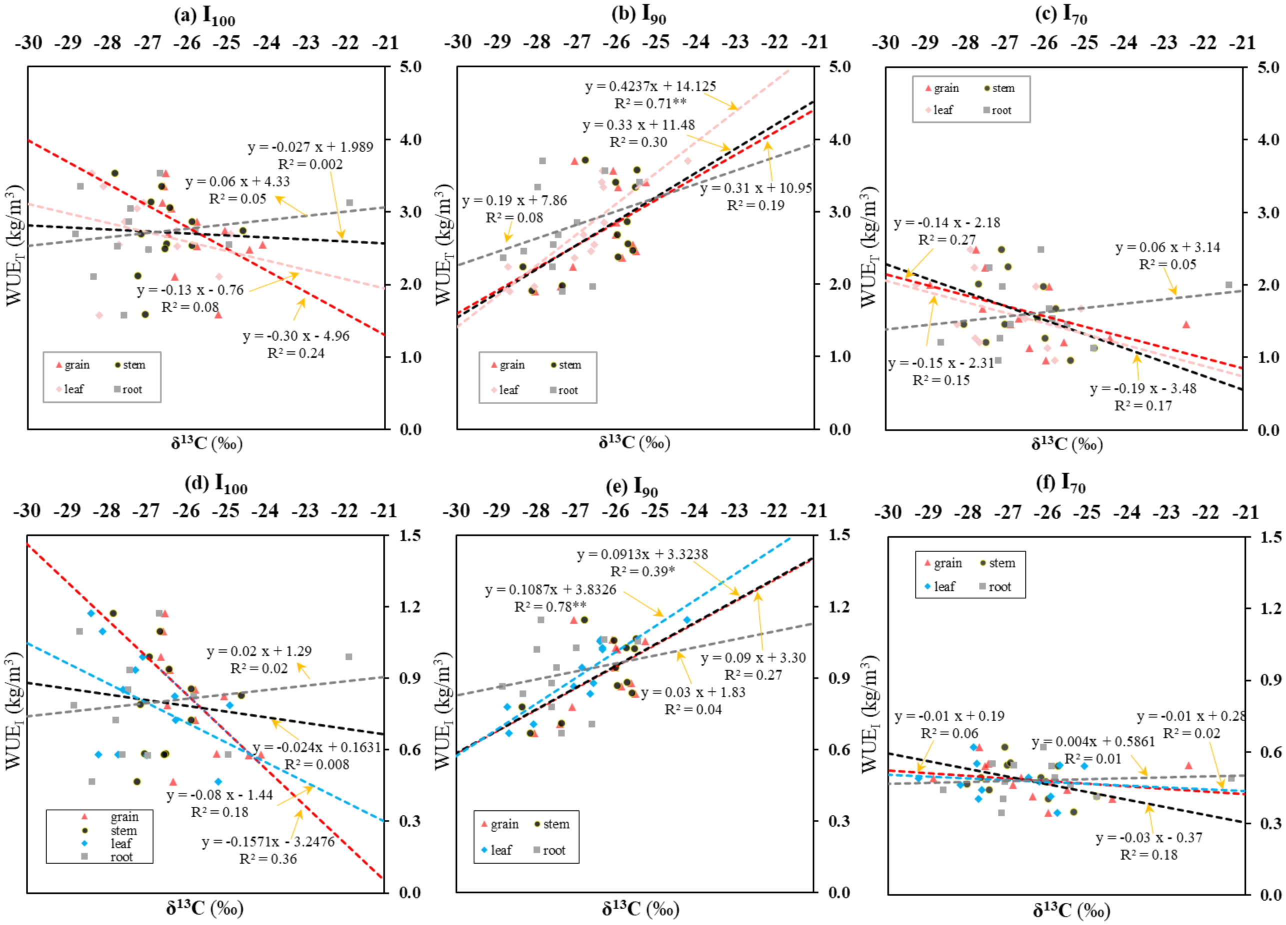

Supplementary Fig. 5 Carbon isotope composition of diverse rice organs ($\delta^{13}C_{grain}$, $\delta^{13}C_{stem}$, $\delta^{13}C_{leaf}$, $\delta^{13}C_{root}$) and two kinds of water use efficiency (WUE_T, WUE_I) as influenced by different water regimes. * and ** represent 0.01<*p* <0.05, and *p*<0.01, respectively.

2.2 Supplementary Table

Supplementary Table 1 Pearson correlation coefficients for crack volume (cm^3), carbon concentration([C]%) and C allocation of diverse organs (grain, stem, leaf, and root, %). * and ** represent 0.01<*p* <0.05 and *p*<0.01, respectively.

	Crack volume	$[C]_{grain}$	$[C]_{stem}$	$[C]_{leaf}$	$[C]_{root}$	Grain C allocation	Stem C allocation	Leaf C allocation	Root C allocation
crack volume	1	0.04	-0.12	0.26	-0.05	-0.69**	0.47**	0.81**	-0.20
$[C]_{grain}$		1	-0.40*	-0.21	-0.12	0.36*	-0.31	-0.12	-0.11
$[C]_{stem}$			1	0.27	-0.16	-0.41*	0.73**	-0.13	0.01
$[C]_{leaf}$				1	0.00	-0.52**	0.18	0.55**	0.06
$[C]_{root}$					1	-0.26	-0.27	-0.10	0.76**
Grain C allocation						1	-.0.66**	-0.69**	-0.20
Stem C allocation							1	0.41*	-0.37*
Leaf C allocation								1	-0.34
Root C allocation									1
